# Anti-Leishmanial and Immunomodulatory Effects of Epigallocatechin 3-O-Gallate on *Leishmania tropica*: Apoptosis and Gene Expression Profiling

**Published:** 2019

**Authors:** Morteza SADUQI, Iraj SHARIFI, Zahra BABAEI, Alireza KEYHANI, Mahshid MOSTAFAVI, Maryam HAKIMI PARIZI, Mahdiyeh GHASEMIAN, Mehdi BAMOROVAT, Fatemeh SHARIFI, Mohammad Reza AFLATOONIAN, Fariba SHARIFIFAR, Pooya GHASEMI NEJAD, Ahmad KHOSRAVI, Ehsan SALARKIA, Rajender S. VARMA

**Affiliations:** 1. Department of Medical Parasitology and Mycology, School of Medicine, Kerman University of Medical Sciences, Kerman, Iran; 2. Leishmaniasis Research Center, Kerman University of Medical Sciences, Kerman, Iran; 3. Pharmaceutics Research Center, Institute of Neuropharmacology, Kerman University of Medical Sciences, Kerman, Iran; 4. Research Center of Tropical and Infectious Diseases, Kerman University of Medical Sciences, Kerman, Iran; 5. Herbal and Traditional Medicines Research Center, Department of Pharmacognosy, Kerman University of Medical Sciences, Kerman, Iran; 6. Regional Center of Advanced Technologies and Materials, Faculty of Science, Palacký University in Olomouc, Šlechtitelů 27, 783 71 Olomouc, Czech Republic

**Keywords:** Epigallocatechin 3-O-gallate, *Leishmania tropica*, Immunomodulatory role, Apoptosis, Gene expression

## Abstract

**Background:**

Pentavalent antimonials such as meglumine antimoniate (MA, Glucantime), are the first-line treatment against leishmaniasis, but at present, they have basically lost their efficacy. This study was aimed to explore epigallocatechin 3-O-gallate (EGCG), alone or in combination with MA against *Leishmania tropica* stages.

**Methods:**

All experiments were carried out in triplicate using colorimetric assay, macrophage model, flow cytometry and quantitative real-time PCR. This experimental study was carried out in 2017 in Leishmaniasis Research Center, Kerman University of Medical Sciences, Kerman, Iran.

**Results:**

Promastigotes and amastigotes were more susceptible to EGCG than MA alone, but the effect was more profound when used in combination. EGCG exhibited high antioxidant level with a remarkable potential to induce apoptosis. Furthermore, the results showed that the level of gene expression pertaining to Th-1 was significantly up-regulated (*P*<0.001).

**Conclusion:**

EGCG demonstrated a potent anti-leishmanial effect alone and more enhanced lethal activity in combination. The principal mode of action entails the stimulation of a synergistic response and up-regulation of the immunomodulatory role towards Th-1 response against *L. tropica.*

## Introduction

Leishmaniasis is a neglected tropical and subtropical complex disease caused by over 20 species of the genus *Leishmania* (1). Cutaneous leishmaniasis is the most frequent type which consists of approximately 70%–75% of the overall reported cases (2). The magnitude of the disease burden due to the chronic conflict in the Middle East countries has significantly been increased by a factor of 6 to 10. Consequently, CL represents a major and large-scale global health challenge (3).

Biological control measures of numerous vectors and reservoirs are impossible and there is neither efficacious vaccine nor effective drugs against all forms of leishmaniasis available universally. Pentavalent antimony compounds (SbV) such as meglumine antimoniate (Glucantime) are the first-line of therapy and are widely used drugs over the last seven decades, which have significantly lost their efficacy. Moreover, the choice of other chemical alternatives for the same reasons is extremely limited. In fact, all of these synthetic chemicals have insufficient action and emergence of disease is a common phenomenon (4,5).

Antioxidants are collectively a diverse group of compounds, including natural products, which possess beneficial health effects. Limitation for insufficient action of SbV emphasizes an urgent need for new additive natural products, combinatory medicines or new treatment modalities to be used as alternative medicines against leishmaniasis (6,7)^.^ Medicinal plants, vegetables, fruits, polyphenols and many other plant extracts possess antioxidant properties that are effective against the infectious agents, degenerative diseases, cancer, neurologic and cardiovascular disorders, and reactive oxygen species (ROS) generated as the result of oxidative stress in normal metabolic processes. Phenolic flavonoids such as green tea consist a wide spectrum of antimicrobial (against bacteria, viruses and fungi) and antiparasitic activities with diverse ranges of therapeutic potentials (8).

The existence of the basic effector Th-1 and Th-2 subtypes of T-lymphocytes (CD4) is now well accepted, and is being used to design therapeutics (drugs) and prophylactic (vaccine) strategies. Although the killing mechanism of the host cells is so complex and multifactorial, the functions of Th-1 and Th-2 cells correlate well with their distinctive cytokine pattern (9). Th-1 is basically involved in cell-mediated delayed-type hypersensitivity (DTH) response and provides killing mechanism against the leishmanial stages which ultimately can become suppression. On the other hand, Th-2 cytokines encourage antibody production, are commonly found in association with strong antibody and allergic reactions and eventually are responsible for the progression of parasite infection (10).

So far, no study has been carried out to explore the effect of the major component of green tea, epigallocatechin 3- O-gallate (EGCG) on the etiological agents of CL in the Old World. EGCG is the most abundant, widely used and the most effective constituent of green tea with great anticancer properties (11,12) and potent leishmanicidal activities against leishmanianiasis in the New World species (13–16) which possesses a broad range of antimicrobial effects (17).

In addition, this study was primarily designed to evaluate the leishmaniacidal effect of EGCG and its antioxidant level, cytotoxic index, apoptotic values and potentials to express distinct gene profiling, alone or in combination with Glucantime, the drug of choice against various types of leishmaniasis, including the mechanism of action. We believe such a broad range of experimental sets performed here are unique and able to elucidate the potential effects of the active constituent of green tea, EGCG on the *L. tropica* pro-mastigote and amastigote stages. This investigation could also be used as a model for the *Leishmania* species in the Old World.

## Materials and Methods

### Parasite and macrophage cell-line

This experimental study was carried out in 2017 in Leishmaniasis Research Center, Kerman University of Medical Sciences. The standard strain of *L. tropica* promastigotes (MHOM/IR/75/Mash2) were seeded in RPMI 1640 medium, supplemented with 100 IU penicillin/mL, 100 μg streptomycin/mL, 10%(v/v) fetal calf serum (FCS) at 56 °C for 30 min and allowed to grow at 24 °C±1 °C and pH 7.2 (all were purchased from Sigma, Aldrich, France).

The parasite was diluted in complete medium and counted by a Neubauer chamber. Murine macrophage cell-line (J774-A1; ECACC no. 91051511) was prepared from the Pasteur Institute of Iran (Tehran).

### Reagents

**Fig. 1:**
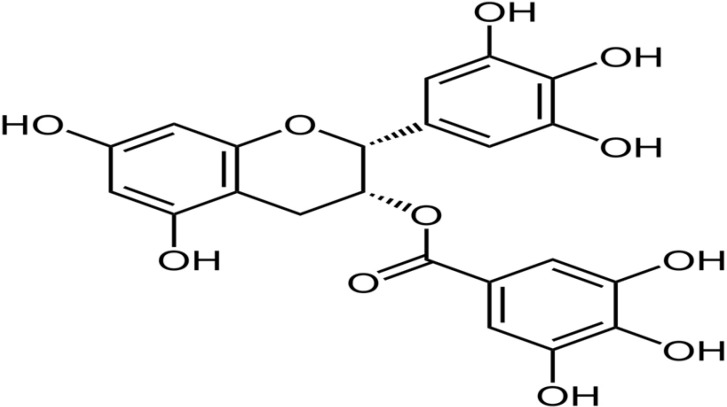
Epigallocatechin-3-O-Gallate Structure, the main component of green tea

Rosewell Park Memorial Institute medium (RPMI 1640), FCS, Pen/Strep, MTT (3-(4,5-dimethylthiazol-2-yl)-2,5-diphenyltetrazolium bromide, a tetrazole), DPPH (zinc sufate, 2,2-diphenyl-1-picrylhydrozy), BHA (butylated hydroxyanisole) and epigallocatechin 3-O-gallate (EGCG) (Fig.1) were purchased from Sigma-Aldrich, France. Glucantime (meglu-mine antimoniate) was kindly provided by the Provincial Health authorities in Kerman University of Medical Sciences originally purchased from Sanofi-Aventis, France.

### Anti-promastigote assay

The activity of EGCG, Glucantime alone or in combination, by a standard colorimetric cell viability MTT assay for determining the susceptibility of promastigotes was evaluated (18). Various dilutions of EGCG or Glucantime (25, 50, 100, 200, 400 and 500μg/mL) and the same serial concentrations for combination of EGCG plus Glucantime in RPMI 1640 (pH 7.2) in 96-well microplate, were prepared. Logarithmic-phase promastigotes (2x10^6^) were harvested from the culture medium and incubated at 24 °C±1 °C for a standard time of 72 h. Promastigotes were cultured in complete medium with no EGCG or Glucantime used as the untreated control group and complete medium with no parasites as the blank. All experiments were repeated thrice (19). The experimental reactions were stopped by isopropanol alcohol and read by spectrophotometer at 492 nm (BioTek-ELx800 Winooski, Vermont, USA). The IC_50_ value (50% inhibitory concentration) was calculated by the probit test.

### Anti-intramacrophage amastigote assay

The stationary phase promastigotes of *L. tropica* was washed with complete RPMI 1640 medium and counted. These metacyclic promastigotes were added to the murine macrophage cell-line at a ratio of 8:1, respectively. The mixtures were then incubated in complete medium at a concentration of 2x10^6^ cells/ml in Lab-Tek 8-chamber microscope slides (0.4 ml/well) at 37 °C for 4 h, in a humid chamber and 5% CO_2_. After removing the free cells by successive washing, the macrophages (containing amastigotes) were adhered to the slides and treated accordingly as mentioned for anti-promastigote assay. All experiments were repeated in triplicate (19) and amastigotes in 100 macrophages per replicate (an overall 300 cells) were counted.

### Antioxidant DPPH assay

The antioxidant level of EGCG was determined according to the method introduced elsewhere. (20). In brief, this method measured the ability of EGCG to scavenge DPPH free radical (21). Any decrease in the DPPH mixture absorbance displayed an increase in the radical scavenging activity was calculated by the following equation: (22) % DPPH radical scavenging = control absorbance-sample absorbance/control absorbance X100. The DPPH alone was used as control and BHA as standard (23).

### Flow cytometry assay

To determine the apoptotic values, an Apoptosis Detection Kit was used according to the manufacture's instruction. Briefly, binding of annexin V/Dead Cell Apoptosis Kit with FITC annexin V and 7AAD, for flow cytometry to promastigotes, was evaluated. The serial dilutions of EGCG and Glucantime were prepared on the day of experimentation as previously mentioned. The anti-promastigote treatments were incubated at 24 °C±1 °C for 72 h, and the microtubes were washed three times with PBS and incubated in dark at ambient temperature. The final analysis was made with Cell Quest software. The externalization of phospholipid classes was the index of leishmanicidal potential as expressed for EGCG compared to Glucantime as positive control. Since the promastigotes of *Leishmania* species lack any detectable amount of phosphotidylserine (PS), we identified other candidate lipid complexes (24).

### Cytotoxicity assay

To evaluate the cytotoxic effects of EGCG and Glucantime on the murine macrophages cell-line, the CC_50_ (cytotoxicity concentration for 50% of cells) of various concentrations were determined. The experimental treatments were then incubated at 37 °C in 5% CO_2_ for 72 h. All experiments were carried out thrice. The selectivity index (SI) as the measure of safety was calculated using the following equation: SI= CC_50_ / IC_50_ ≥10; non-toxic.

### Quantitative real-time PCR (qPCR) RNA isolation and analysis

Total cellular RNA was extracted using EZ-10 Spin Column Total RNA Miniprep Kit (Bio Basic Inc., Canada) according to the manufacturer’s instructions. The purity and quantity of the extract were determined by Nanodrop ND-1000 spectrophotometer (Thermo Scientific, Wilmington, DE, USA).

### Real-time PCR

Overall, 500 ng RNA was reverse transcribed to cDNA, using Revert Aid M-MuLV reverse transcriptase with a random hexamer primer (both from Fermentas, Vilnius, Lithuania). The sequence of primers and the expected PCR products for β-actin, TATA-binding protein, and cytokine genes are listed in Table 1. Quantitative RT-PCR of target cDNA was conducted on a Rotor gene 6000, Corbett, sequence detection system. The PCR reaction for mRNA detection was carried out in 10 μL reaction volumes possessing 5 μL 2X SYBR Green Supermix SYBR^®^ Premix Ex Taq^TM^ (Takara Bio Inc., Otsu, Japan), 250 nmol from each of forward and reverse primer, and 1 μL cDNA diluted in RNase-free H_2_O.

**Table 1:** Primers used for quantitative real-time PCR

***Template***	***Forward primer(5′-3′)***	***Reverse primer(5′-3′)***
IL-12	TGGAGTGCCAGGAGGACAGT	TCTTGGGTGGGTCAGGTTTG
IL-1β	ACAGATGAAGTGCTCCTTCCA	GTCGGAGATTCGTAGCTGGAT
Meta caspase	CAGCAACAATTCCTGGCGATA	AAGTTTGAAGTAAAAGGAGACAATTTGG
iNO	AACAGCCTCACAGAGCAGAAGAC	GCCCTGCAGAAGGTTTCCTT
IL-10	CACTCCCAAAACCTGCTGAG	TCTCTTCAGAAGTGCAAGGGTA

Two-step PCR conditions were carried out at 95 °C for 30 sec and then 40 cycles at 95 °C for 5 sec and 60 °C for 30 sec.

Each set of reaction also included negative (i.e., water instead of sample) and RT (samples containing RNA, which was not reverse transcribed) controls. For each gene, PCR reactions were performed in duplicate. PCR results were optimized to the levels of β-actin and HPRT genes as reference. DCt was calculated using the following formula: [ΔCT = CT (target) _ CT (reference)]. Gene expression level was determined by 2 ^−ΔCt^ method. Fold increase (FI) was calculated using the comparative threshold method (2 ^−ΔΔCT^).

### Statistical analysis

The data analyses were performed using SPSS package ver. 20 (Chicago, IL, USA). Differences between control groups or among various concentrations of EGCG or Glucantime were carried out by analysis of variance (ANOVA) and Student^’^s *t*-test. The IC_50_ and CC_50_ values were calculated using the probit test in SPSS. The data are given as the mean ± standard deviations (SD). The results were statistically defined significant at *P*<0.05.

## Results

### Anti-promastigote effect EGCG and Glucantime alone

**Fig. 2:**
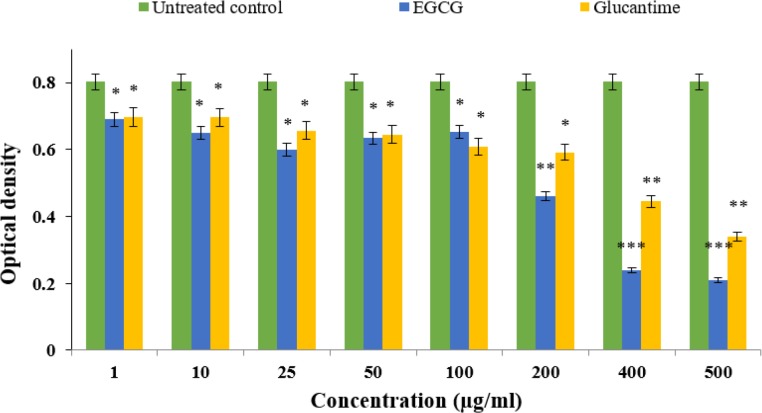
Comparison of the optical density (OD) values between Epigallocatechin 3 – O – gallate (EGCG) or Glucantime alone and untreated control (UC), on the susceptibility of promastigotes treated with various concentrations by colorimetric assay (MTT). (^***^P<0.001, ^**^P<0.01, ^*^P<0.05)

**Fig. 3:**
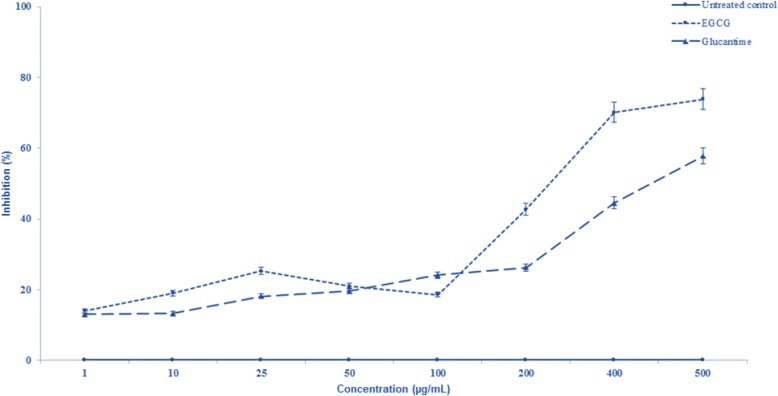
Inhibition level of Epigallocatechin-3-O-Gallate on promastigotes of *Leishmania tropica* compared to Glucantime as positive control. Data are mean ± SD of triplicate experiments

The OD values for EGCG and Glucantime as measured by ELISA were significantly lower compared to the negative control (*P*<0.001 vs. *P*<0.005, respectively; Fig. 2). Although the reduction rate was significantly different at 200 μg/ml concentration of EGCG, at higher concentrations (400 and 500 μg/ml) displayed similar activity compared to the untreated control (*P*<0.001). Both EGCG and Glucantime significantly inhibited (*P*<0.001) *L. tropica* promastigote proliferation rate at various concentrations following 72 h of standard incubation period compared to the untreated control group (Fig. 3). The inhibitory activity displayed a dose-dependent pattern, that is, the effect was more profound at higher concentrations of EGCG (200, 400 and 500 μg/ml) compared to the untreated control. Interestingly, EGCG demonstrated a superior inhibitory effect than Glucantime as the positive control drug.

### EGCG and Glucantime in combination

The combinatory effect of EGCG and Glucantime on *L. tropica* promastigote was more potent compared to each compound alone (25–500 μg/ml) for 72 h.

**Fig. 4:**
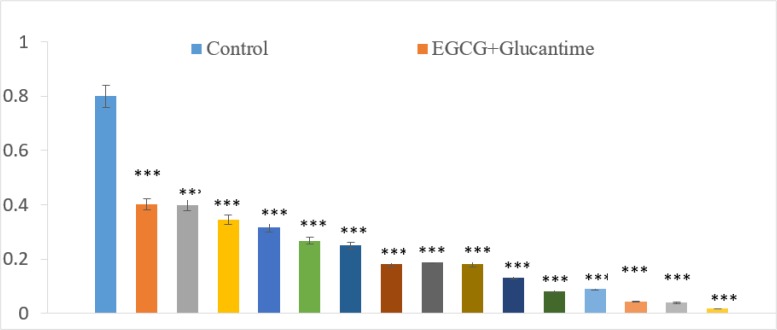
The optical density (OD) of *L. tropica* promastigotes decreased significantly (^***^P<0.001) at higher concentrations. The data are presented as the means ± SD of triplicates

This combination decreased *L. tropica* promastigotes viability in a dose-dependent response (*P*<0.001). The inhibitory effect was <1% when EGCG combined with Glucantime at 500 μg/ml, each (Fig. 4).

### Anti-intramacrophage amastigote effect EGCG and Glucantime alone

The effect of EGCG on intramacrophage amastigotes at different concentrations was assessed and compared to Glucantime as the positive control group (Table 2). Both, EGCG and Glucantime decreased *L. tropica* amastigotes viability in a dose-dependent effect when compared to the negative control group (*P*<0.001); although, an exception being for EGCG which its effect was slightly greater at 25 μg/ml than the corresponding dose for Glucantime (*P*<0.001 vs. *P*<0.005, respectively). The activity was higher when the mean number of amastigotes treated with EGCG or Glucantime was evaluated (n= 23.03±2.3 vs. 46.83±2.9, respectively).

**Table 2:** The effect of various concentrations of Epigallocatechin-3-O-Gallate (EGCG) and Glucantime alone on the mean number of amastigotes in each macrophage x100 in triplicate. The data are presented as the means ± SD of triplicates

***Concentration (μg/mL)***	***EGCG*** ***Mean ± SD***	**P *value*** **P *value***	***Glucantime*** ***Mean ± SD***	**P *value*** **P *value***
0 (Untreated control)	84.2 ± 1.8	NR^*^	84.2 ± 1.8	NR^*^
25	35.4 ± 1.7	0.001	75.3 ± 2.1	0.005
50	32.8 ± 2.4	0.001	67.1 ± 1.4	0.001
100	28.3 ± 1.8	0.001	55.5 ± 1.6	0.001
200	22.3 ± 2.3	0.001	42.7 ± 2.5	0.001
400	14.5 ± 2.0	0.001	28.3 ± 2.0	0.001
500	4.9 ± 0.2	0.001	12.1 ± 0.9	0.001
Mean	23.03±2.3	0.001	46.83±2.9	0.001

* NR: Not Related

### EGCG and Glucantime in combination

Similar to promastigotes, both combination of EGCG and Glucantime significantly (*P*<0.001) decreased the multiplication rate of intramacrophage amstigotes in a dose-dependent manner (Table 3).

**Table 3:** The effect of various concentrations of Epigallocatechin-3-O-Gallate combined with Glucantime on the mean number of amastigotes in each macrophage x100 in triplicates. The data are presented as the means ± SD of triplicates

***Concentration (μg/mL)***	***EGCG+ Glucantime***		***Concentration***	***EGCG+ Glucantime***	
	***Mean***	**P *value***	***(μg/mL)***	***Mean***	**P *Value***
0 (Untreated Control)	100.8±2.1	NR	200+400	19.95±1.6	0.001
50+200	31.5±2.4	0.001	200+500	19.1±1.9	0.001
50+400	30.7±1.9	0.001	400+200	14.3±1.5	0.001
50+500	30.1±1.8	0.001	400+400	13.55±2.0	0.001
100+200	28.45±2.0	0.001	400+500	12.95±1.9	0.001
100+400	27.65±1.7	0.001	500+200	4.6±1.4	0.001
100+500	26.9±2.1	0.001	500+400	4.25±1.8	0.001
200+200	21.35±2.2	0.001	500+500	3.95±0.8	0.001

Comparison of the IC_50_ values for promastigotes and amastigotes indicated that both stages were significantly more susceptible to EGCG than Glucantime (*P*<0.001); although, the values for promastigotes and amastigotes treated with EGCG were 6.6 and 8.3 fold lesser than the corresponding stages for Glucantime (Table 4).

**Table 4:** Comparison of the mean IC50 values (μg/ml) of *Leishmania tropica* promastigote and amastigote stages treated with various concentration of Epigallocatechin-3-O-Gallate, Glucantime and cytoxicity effect of EGCG and Glucantime

***Compound***	***CC_50_ (μg/mL)*** ***Macrophage***	***IC_50_ (μg/mL)*** ***Amastigote***	***IC_50_ (μg/mL)*** ***Promastigote***	***SI***
EGCG	283.40 ± 0.065	20.65 ± 0.087	86.96 ± 0.077	13.72
Glucantime	1867.26 ± 0.072	172.23 ± 0.093	1225 ± 0.140	10.84

Selectivity index (SI) = (CC_50_/IC_50_),SI ≥ 10

### Antioxidant activity

**Fig. 5:**
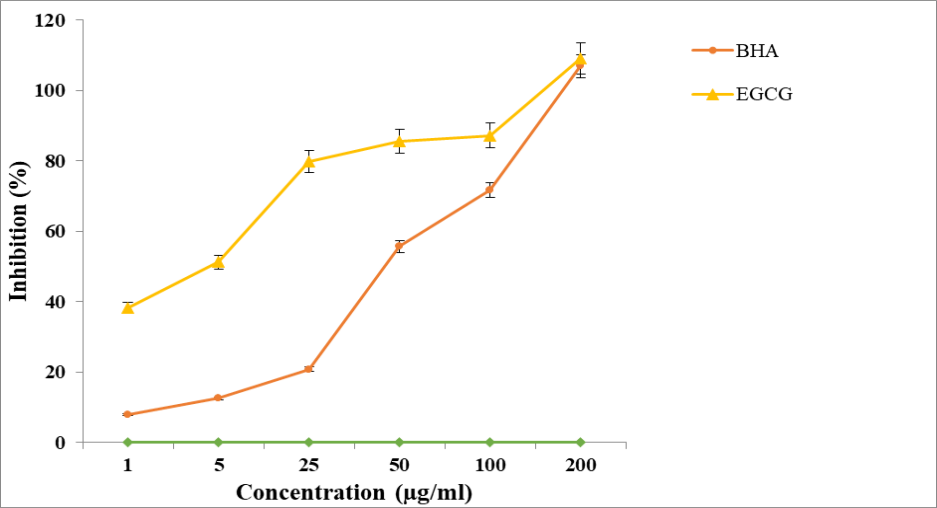
Scavenging effects of Epigallocatechin-3-O-Gallate (EGCG) on 1,1-diphenyl-2-picrylhydrazyl (DPPH) free radicals compared to butylated hydroxyanisole(BHA) as a standard control. Data are means ± SD of trip-licate experiments.

The antioxidant level of EGCG in comparison to the standard control (BHA) was evaluated (Fig. 5). The activity followed a dose-response effect and the overall antioxidant level was 16.5 times higher (*P*<0.001) than the standard control (IC_50_ = 2.93 μg/ml vs. IC_50_ = 48.3 μg/mL).

### Apoptotic analysis

**Fig. 6:**
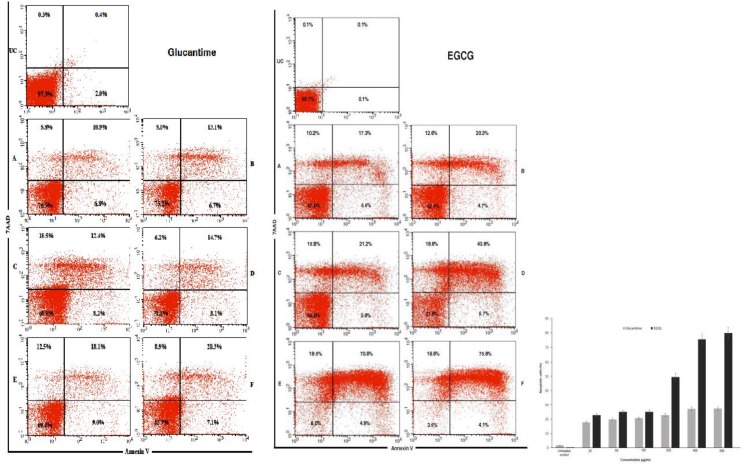
Apoptotic profiles of *Leishmania tropica* promastigote with annexin V at various concentration of Epigallocatechin-3-O-Gallate (EGCG) or Glucantime alone. A: 25μg/mL; B: 50μg/mL; C: 100μg/ml; D: 200μg/mL; E: 400μg/mL; E: 500μg/mL; UC: Untreated control

The apoptotic values as measured by flow cytometry analysis exhibited significant levels of inhibitory effects (*P*<0.001); by increasing the concentrations of EGCG from 25 μg/mL to 500 μg/ml, the apoptotic values increased after 72 h of incubation. The programmed cell death (PCD) increased extensively in a dose-response manner from 25% at 50 μg/ml to 79.9% at 500 μg/mL (Fig. 6).

### Cytotoxicity effect

The cytotoxic effect of EGCG and Glucan-time on macrophages as harboring cells was determined by MTT cell viability experiments and the OD values form the basis for calculating the CC50 levels. Then based on the following equation the selectivity index as the measure of cytotoxicity was calculated. EGCG and Glucantime similarly demonstrated a safety index of 13.72 and 10.84 μg/ml, respectively (Table 4).

### Gene expression level

**Fig. 7:**
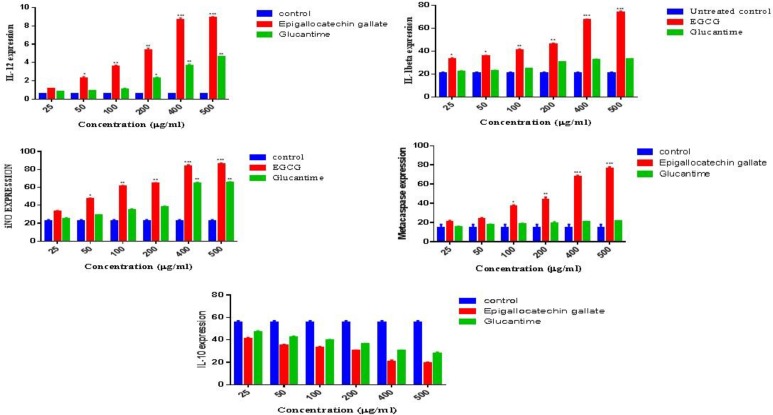
Gene expression profiles of IL-12p40, IL-1beta, Metacaspase, iNO and IL-10 on *Leishmania tropica* promastigtes treated by Epigallocatechin-3-O-Gallate or Glucantime compared with untreated control (*P*<0.001) as measured by quantitative real-time PCR.

The gene expression levels of interleukin-12 (IL-12 p40), IL-1 beta, inducible nitric oxide synthase (iNOS gene) as the measure of Th-1 significantly up-regulated (*P*<0.001) and meta-caspase as well, whilst the level of IL-10 as the indicator of Th-2 was significantly down-regulated (*P*<0.001) from 25 μg/ml to 500 μg/mL (Fig. 7).

## Discussion

Epigallocatechin 3-O-gallate is the main component of green tea commonly used and possesses variable ranges of therapeutic effects against infectious and non-infectious diseases (13,17,25,26). In the present study EGCG and Glucantime alone or in combination displayed pronounced levels of antileishmanial effect against *L. tropica* promastigote and intramacrophage amastigote stages on in vitro models.

The selectivity index (SI) as the measure of toxicity for the treatment of macrophages with EGCG or Glucantime resulted in 13.72 μg/ml and 10.84 μg /ml at 72 h, respectively. The biological activity of a compound is not associated to cytotoxicity when SI ≥10 (27). These results exhibited a greater safety index for EGCG compared to Glucantime. The results found here are also consistent with those performed on *L. braziliensis* (28) and *L. amazonensis* stages (15) in the New World. Amastigotes were more susceptible to EGCG or SbV than promastigotes, as they can readily reduce SbV to SbIII, whilst promastigote stage cannot (29). Amastigotes have a superior ability to concentrate drugs than promastigotes (30). These two stages are biologically and physiologically different in their susceptibility to oxidative stress intermediates which have largely been the basis of parasitic chemotherapy.

Nitric oxide is highly hydrophobic and diffusible and partitions readily into cell membranes affecting the cellular organelles accordingly (31). A number of drug models such as paraquat, quercetin, staurosporine, menadione and curcumin among others, also rely on ROS generation as the main mode of action against the common parasites (32). Chalcones inhibit fumarate reductase leading to alterations in the function and ultrastructure of the mitochondria, and ultimately contributing to killing the parasite (33–36). Phagocytic cells including macrophages are estimated to produce a considerable amount of ROS including oxygen superoxide (O_2_.^−^), NO, H_2_O_2_ and varying ranges of proteolytic enzymes; each of which acts as a powerful tool to combat the invading organisms. These molecules such as H_2_O_2_ are released into the vacuoles from the cytoplasmic granules to produce hypochlorous acid (HOCL), a potent anti-infectious agent (8).

Employing respiratory chain inhibitors (32), showed compelling evidence for the generation of ROS by complexes I, II and III. The ROS generated by inhibition of complex II led to increased levels of intracellular ca^2+^ which, in turn, resulted in cellular apoptosis. EGCG induces iNOS gene transcription and cytokines production as documented by gene expression of iNOS in this study and in various biological processes (37,38). Therefore, we studied whether EGCG mediates iNOS and cytokines induction in *L. tropica* as a possible mode of action contributing to cell death. In addition, we targeted IL-10 as the measure of Th-2 response. EGCG treatments for 72 h greatly increased IL-12, IL-1ß, metacaspase and also iNOS expression. In contrast, the induction of IL-10 gene expression was suppressed as clearly demonstrated by the present study. Metacaspases are cysteine peptidases in eukaryotes and plants as well. Several role models including cell cycle control, cell death or even cell survival have been proposed. The role of *L. major* metacaspase in the cell death and autophagy pathway as well; this may reflect that different protein domain are involved (39).

There is a synergistic effect between EGCG and Glucantime in inhibiting *L. tropica* stages; although, more potent when used in combination, designed to up-regulate the immunomodulatory role against *L. tropica* promastigotes and amastigotes, the causative agent of ACL in the Old World. Therefore, natural plant components such as EGCG provide the rich source of low price and commonly available products with high safety index, which could be the valuable source of essential molecules against CL notably in endemic countries.

## Conclusion

EGCG exhibited a potent antileishmanial effect individually and more potent lethal activity in combination with Glucantime than expected, designed to stimulate a synergistic response leading to up-regulation of the immunomodulatory role against *L. tropica*. Hence, EGCG could potentially be used as a drug model for generating iNOS transcripts and possibly other ROS as the principal mode of action against the leishmanial stages. The role of EGCG as a potent antioxidant in inhibition of promastigotes and amastigotes provides the rationale for immunomodulatory therapy to potentiate and shift the immune response towards Th-1 to generate regulatory mechanisms as demonstrated in the present study. Therefore, further studies are highly desirable to evaluate its efficacy in clinical settings against CL.
